# P-900. Demonstrating Your Expertise: Advanced Leadership Certification in Infection Prevention

**DOI:** 10.1093/ofid/ofae631.1091

**Published:** 2025-01-29

**Authors:** Jessica Dangles, Mayar Al Mohajer

**Affiliations:** Certification Board of Infection Control and Epidemiology, Arlington, Virginia; Baylor College of Medicine, Houston, TX

## Abstract

**Background:**

The Certification Board of Infection Control and Epidemiology (CBIC) developed the Advanced Leadership Certification in Infection Prevention (AD-CIP). This portfolio-based assessment indicates professional expertise and leadership in infection prevention and control and healthcare epidemiology. This longitudinal perspective provides valuable insights into an applicant’s achievement beyond a single snapshot assessment.

**Methods:**

A group of ten IPC subject matter experts (SMEs), along with a psychometric consultant, applied principles of measurement theory and assessment design to ensure the reliability, validity, fairness, and practicality of the assessment. The SMEs met via focus groups to conduct a job task analysis (JTA) of advanced IPC professionals' job roles and responsibilities. The JTA determined the competency areas essential to advanced leadership within IPC.

**Results:**

The resulting framework targeted specific learning outcomes guided by an existing competency model developed by the Association for Professionals in Infection Control and Epidemiology. Clear guidelines for collecting, organizing, and evaluating portfolio submissions within the scoring rubric were required to establish scoring procedures. Inter-rater reliability analysis was used to assess scoring consistency among multiple raters. A pilot test of the assessment was conducted to identify any issues with the assessment design, instructions, or scoring procedures. The final assessment measures two domains within IPC: leadership and professionalism. Within each domain are subdomains of practice and innovation, change management, quality improvement, and advocacy. Applicants are required to submit supporting evidence indicating how the criteria for each domain and subdomain are met.

**Conclusion:**

IPC leaders use their skills to establish a clear vision and strategic direction, facilitate change that improves programs and practices, enhance individual and population health, and reduce the risk of infection across the lifespan in all settings. The AD-CIP allows IPC professionals to demonstrate how their leadership has a measurable impact on the profession.

**Disclosures:**

**All Authors**: No reported disclosures

